# Eye movements during path integration

**DOI:** 10.14814/phy2.13921

**Published:** 2018-11-18

**Authors:** Jan Churan, Anna von Hopffgarten, Frank Bremmer

**Affiliations:** ^1^ Department of Neurophysics Philipps‐Universität Marburg Marburg Germany; ^2^ Center for Mind, Brain and Behavior Philipps‐Universität Marburg Marburg Germany

**Keywords:** Auditory, distance reproduction, Eye movements, multi‐modal, self‐motion, visual

## Abstract

Self‐motion induces spontaneous eye movements which serve the purpose of stabilizing the visual image on the retina. Previous studies have mainly focused on their reflexive nature and how the perceptual system disentangles visual flow components caused by eye movements and self‐motion. Here, we investigated the role of eye movements in distance reproduction (path integration). We used bimodal (visual‐auditory)‐simulated self‐motion: visual optic flow was paired with an auditory stimulus whose frequency was scaled with simulated speed. The task of the subjects in each trial was, first, to observe the simulated self‐motion over a certain distance (Encoding phase) and, second, to actively reproduce the observed distance using only visual, only auditory, or bimodal feedback (Reproduction phase). We found that eye positions and eye speeds were strongly correlated between the Encoding and the Reproduction phases. This was the case even when reproduction relied solely on auditory information and thus no visual stimulus was presented. We believe that these correlations are indicative of a contribution of eye movements to path integration.

## Introduction

Movement of an observer through an environment generates a visual flow field on the retina. The properties of this flow field were shown to provide important cues for the estimation of parameters of self‐motion, such as direction (heading), speed, and traveled distance, i.e., path integration (Gibson 1950; Andersen et al. 1993; Lappe and Rauschecker 1994; Lappe et al. 1996, 1999; Bossard et al. 2016). Radial optic flow elicits a nystagmus‐like pattern of slow and fast conjugate eye movements (Niemann et al. 1999). One function of these eye movements is to stabilize the visual image on the retina and thereby improve the visibility of potential targets (Warren and Rushton 2009). On the other hand, the extraction of self‐motion information from optic flow is complicated by these eye movements which by themselves induce large‐scale shifts of the retinal image (Warren and Hannon 1990; Bremmer et al. 2017). Thus, successful navigation critically depends on the ability to distinguish those components of the optic flow that are caused by self‐motion from those caused by eye movements (Royden et al. 1992, 1994; for a review see Angelaki and Hess 2005).

It is well‐known, that path integration, i.e., the extraction and integration of motion information to guide spatial behavior, involves sensory and motor processing (Etienne and Jeffery 2004). On the sensory side, visual (Bremmer and Lappe 1999; Lappe et al. 2007), auditory (von Hopffgarten & Bremmer 2011), tactile (Churan et al. 2017), and vestibular (Berthoz et al. 1995; Glasauer et al. 2002) signals have been unequivocally shown to contribute to estimating traveled distance. On the motor side, as an example, efference copy signals of walking have been implicated in path integration behavior (Mittelstaedt and Mittelstaedt 2001). While eye movements as reactions to an actual visual flow field are well described (e.g., Lappe et al. 1998; Angelaki and Hess 2005; Bremmer 2011); not much is known on how they contribute to behavioral tasks like path integration.

In our experiments, we investigated eye movements during a distance reproduction task (von Hopffgarten & Bremmer 2011) based on visual and/or auditory cues. In natural environments, auditory cues can provide information about self‐motion by changes e.g., of loudness, the Doppler effect or interaural time differences (Lutfi and Wang 1999). Here, we used differences in the frequency of a tone as an indicator of the speed of self‐motion. This association was rather learned than natural – although it occurs frequently, for example during operation of motor powered vehicles. The task of the subjects was (1) to monitor the distance of a simulated self‐motion based on visual and auditory cues (Encoding phase) and then (2) to reproduce the perceived distance actively while using only auditory, only visual or bimodal (visual‐auditory) information (Reproduction phase). Like during real self‐motion, subjects were free to move their eyes in both, the Encoding and the Reproduction phase. We focused on analyzing the role of slow eye movements, since fast eye movements recently have been shown to compromise encoding of (visually simulated) self‐motion (Bremmer et al. 2017). In particular, we were interested in the oculomotor behavior during the Reproduction phase when no visual stimulus was available, and the subjects had to rely only on auditory information.

## Methods

Behavioral aspects of this study concerning the ability of participants to reproduce travel distance have been published before (von Hopffgarten & Bremmer 2011). Here, we focused on the oculomotor behavior of the subjects from the same dataset, which was not analyzed in this previous work.

### Subjects

Six human subjects (two male and four female, mean age 23 years) took part in the experiment. The subjects had normal or corrected‐to‐normal vision and normal hearing. All subjects gave informed written consent prior to the testing and were reimbursed after the experiment with 8 €/h. All procedures in this study conformed to the Declaration of Helsinki and were approved by a local ethics committee.

### Apparatus

Experiments were conducted in a darkened (but not completely dark) sound attenuated room. Subjects were seated at a distance of 114 cm from a tangential screen (70° x 55° visual angle) and their head‐position was stabilized by a chin‐rest. Visual stimuli were generated on a windows PC using an in‐house built stimulus package and were back‐projected onto the screen by a CRT‐Projector (Electrohome Marquee 8000) at a resolution of 1152 x 864 pixels and a frame rate of 100 Hz. The auditory stimuli were also generated using MATLAB and presented to the subjects by head‐phones (Philips SHS390). The eye position was recorded by a video‐based eye‐tracker (EyeLink II, SR Research) at a sampling rate of 500 Hz and an average accuracy of ~0.5°. During the distance reproduction, the subjects controlled the speed of simulated self‐motion using an analog joystick (Logitech ATK3) that was placed on a desk in front of them. The speed of the simulated self‐motion was proportional to the inclination angle of the joystick. The data from the joystick were acquired at a rate of 100 Hz and minimal change in speed of simulated self‐motion that could be triggered by the joystick was 1/1000 of the maximum range of speeds used in the experiments.

### Stimuli

**Figure 1:**
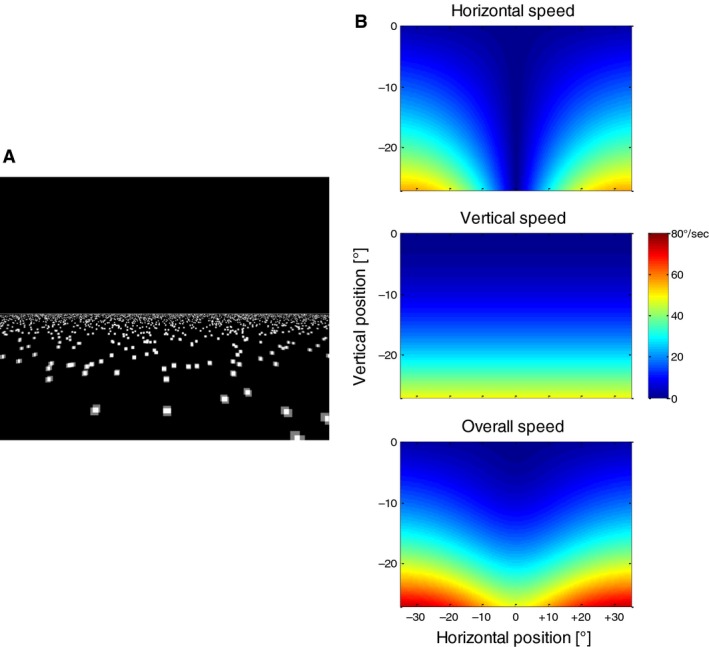
(A) Screenshot of the stimulus simulating self‐motion across a ground plane of randomly placed squares. (B) Distribution of visual speeds in different parts of the optic flow pattern. Note that only the lower half of the screen is depicted in the colored displays since no stimulus was present in the upper half.

The visual stimulus consisted of a horizontal plane of white (luminance: 90 cd/m^2^) randomly placed small squares (Fig. 1A) on a dark (<0.1 cd/m^2^) background that filled the lower half of the screen. The size of the squares was scaled between 0.2° and 1.9° in order to simulate depth. The direction of the simulated self‐motion was always straight‐ahead (orthogonal relative to the orientation of the horizon within the stimulus). Since the subjects did not know the absolute size of the squares and the absolute altitude of the observer above the plane, they could not determine the absolute speed of simulated self‐motion from the stimulus display. Thus, here, the distances are always quantified in arbitrary units (AU) and the speed of simulated self‐motion in AU/s. The local horizontal and vertical speeds of the optic flow depended on the position on the screen (Fig. 1B), with lowest speeds close to the focus of expansion (in the center of the screen) and increasing with increasing eccentricity. The auditory stimuli were sinusoidal tones (SPL approximately 80 dB) with a frequency proportional to the simulated speed. The frequencies were in a range between 220 and 440 Hz and changed linearly as a function of the speed of the simulated self‐motion, which was in the range of 0–20 AU/s.

### Procedure

Each trial consisted of two phases. During the “Encoding phase” the subjects were presented with a simulated self‐motion at one of the three speeds (8, 12 or 16 AU/s). The presentation lasted 4 seconds each which resulted in three different traveled distances (32, 48, 64 AU). The presentation was always bimodal, i.e., visual motion was accompanied by a sound representing the respective speed. The sound frequencies corresponding to the three speeds used during the Encoding phase were 308, 354, and 396 Hz, respectively. The task of the subjects in this phase was to monitor the distance covered for later reproduction. After the Encoding phase, a dark screen was presented for 500 msec and then the subjects had to reproduce the previously observed distance using a joystick. In different conditions of this “Reproduction phase,” either only the visual display was presented (visual condition) or only the auditory stimulus was presented while the screen was dark (auditory condition) or both sources of information were available at the same time (bimodal condition). During reproduction, the subjects were able to change the simulated speed by changing the inclination of the joystick. After the subjects had reached the distance they perceived to be identical to that during the Encoding phase, they had to press a joystick button to complete the trial. The subjects were allowed to move their eyes freely during the Encoding and the Reproduction phases. There were thus nine different experimental conditions: three different speeds in three different modalities. In each experimental condition, 80 trials were recorded. All conditions were presented in a pseudo‐randomized order and the subjects were not informed in advance about the sensory modality of the Reproduction phase. The duration of the experiment for each subject was ~4 h, including breaks.

### Data analysis

Eye position as well as the speed of the simulated self‐motion were recorded at a sampling rate of 500 Hz. As detailed above, we focused on the slow eye movements in the different experimental conditions. To this end, we removed the parts of the data in which the subjects made saccades, fast resetting eye movements, or blinks. In addition, we removed all other parts of the data in which the eye‐speed exceeded 50°/sec. These de‐saccaded data were used to calculate the average position, speed, and direction of the slow eye movements per trial. All calculations and data transformations were done using MATLAB (The MathWorks). Some statistical analyses were performed using the SPSS package (IBM).

The spatial distribution of eye positions and the relationship between eye position and eye speed were determined for each subject and experimental condition by binning the horizontal and vertical eye positions in bins of 0.1 × 0.1° and then calculating the average horizontal and vertical eye speeds in each bin. We used Pearson correlations to calculate the dependence between position and eye speed; however, the results were not different when a nonparametric measure (Spearman correlation) was used. Preferred eye positions were calculated by fitting a two‐dimensional Gaussian function to the frequency distribution of eye positions by a least squares algorithm.

## Results

### Eye‐position during the encoding‐ and reproduction phases

**Figure 2:**
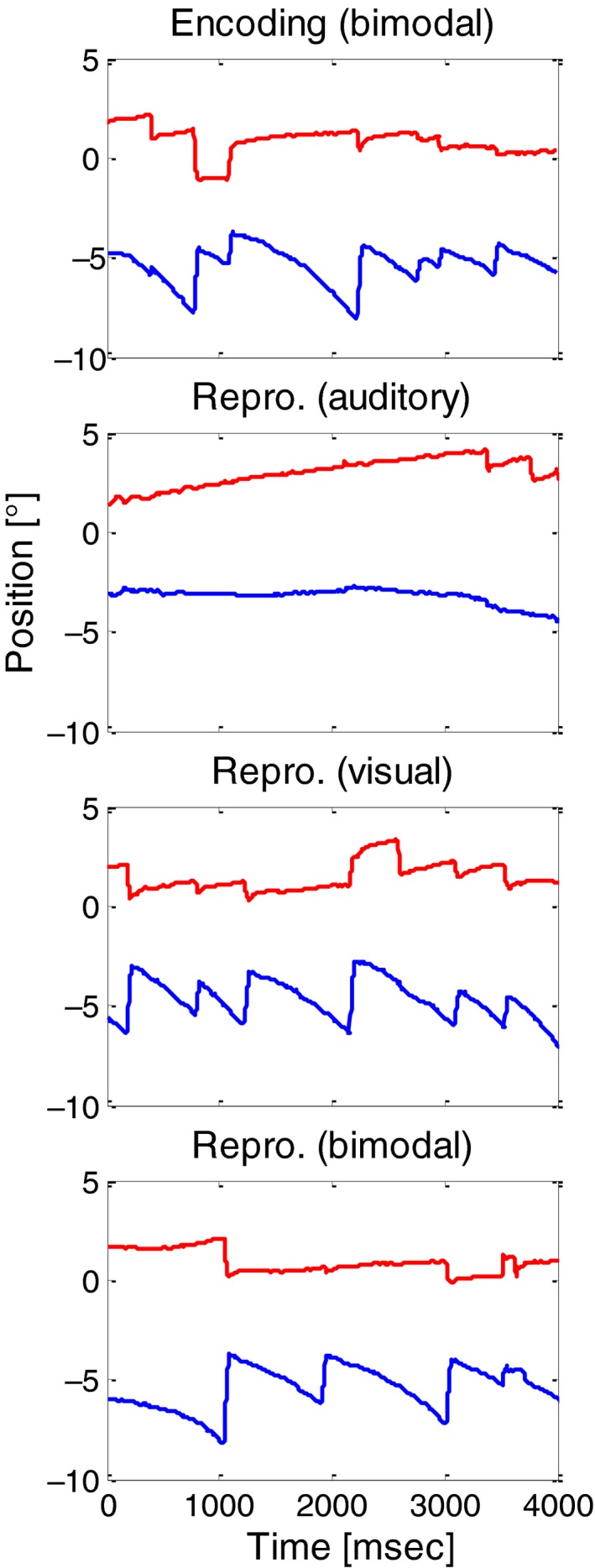
Example horizontal (red) and vertical (blue) eye traces (from subject S4) for the Encoding phase (upper panel) and for the three different modality conditions in the Reproduction phase. The time axes represent the time from the start of the stimulus motion. The speed of the simulated self‐motion was 16 AU/sec.

Figure 2 shows example of eye traces during the Encoding phase (bimodal) and the three different modality conditions of the Reproduction phase. When a visual stimulus was present (visual and bimodal conditions), a nystagmus‐like eye movement pattern emerged consisting of slow eye movements in the direction of the optical flow (mostly vertical) and fast resetting eye movements in the opposite direction. In the purely auditory condition (i.e., on a dark screen), the eye traces revealed mostly prolonged drifts with fewer saccadic eye movements in between.

**Figure 3:**
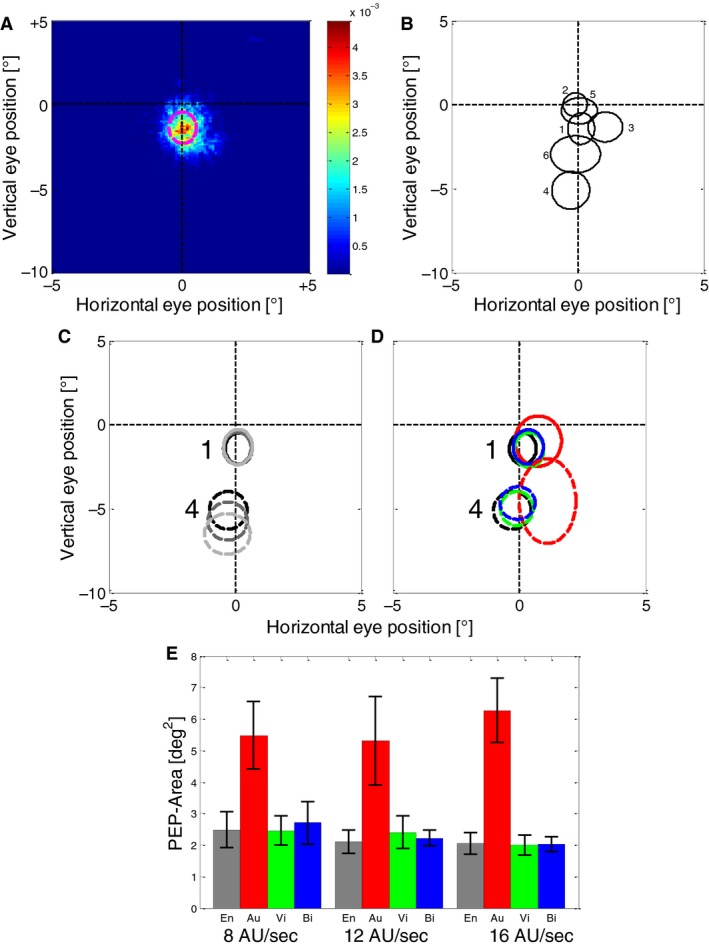
(A) The distribution of eye positions on the screen during the Encoding phase (speed = 16 AU/sec, 240 trials) for another example subject (S1). The colors show the probability of eye position to be in a certain spatial bin with a resolution of 0.1 × 0.1°. The dashed magenta line shows one standard deviation of a 2D Gaussian fitted to the distribution data. Vertical position 0° represents the horizon of the simulated ground plane. Note that the figure does not show the entire screen (that was 70 × 55° large) but only a central part of 10 × 15°. (B) 2D Gaussian fits of position distribution (preferred eye positions, PEPs) of all six subjects in the Encoding phase at a simulated speed of 16 AU/sec. Like in 3A, the lines represent one standard deviation of a 2D Gaussian distribution fitted to the data of each subject. (C) PEPs during the Encoding phase at different speeds for two example subjects (distinguished by different line styles). Light grey lines show the PEPs at a simulated speed of 8 AU/sec, dark grey at 12 AU/sec, and black at 16 AU/s. (D) Comparison of PEPs in the Encoding phase and different modality conditions of the Reproduction phase for two example subjects (the same as in C) at a simulated speed of 16 AU/sec. Data from (i) the Encoding phase (which was always bimodal) is shown in black, (ii) auditory Reproduction in red, (iii) visual Reproduction in green, and (iv) bimodal Reproduction in blue. (E) Means and standard errors of the PEP‐size for all subjects. Different bars show different conditions (combinations of simulated speed, Encoding and Reproduction phase, and modality used in the Reproduction phase). Grey bars represent eye positions during the Encoding phase (bimodal), red bars eye positions during auditory Reproduction (empty screen), green bars during visual Reproduction and blue bars during bimodal Reproduction, respectively.

As shown in Figure 3A for one example subject (S1), eye positions during the Encoding phase covered only a very small portion of the stimulus display. We fitted two‐dimensional Gaussians to the spatial distributions of eye‐position for each subject and condition and found that each subject used a very localized – but also highly individual part of the stimulus to obtain information. An example is shown in Figure 3B for all subjects in the Encoding phase at a simulated speed of 16 AU/s. In the following we name the area within one standard deviation around the center of the Gaussian fit the Preferred Eye Position (PEP). The PEP depended to some extent on the speed of the stimulus – in particular for those subjects that preferred to sample higher stimulus speeds (i.e., positioned their eyes lower on the screen, subject 4 in Fig. 3C). However, significant overall changes in the position of the PEP were not found in the Encoding phase neither in the horizontal (repeated‐measures ANOVA, *F* = 2.61, *P* = 0.12) nor in the vertical (repeated‐measures ANOVA, *F* = 1.21, *P* = 0.32) dimension. The subjects adhered to the PEP they showed during the Encoding phase, even during the Reproduction phase (Fig. 3D). When no visual stimulus was present on the screen (auditory Reproduction phase, red ellipses in Fig. 3D), the PEP was slightly shifted relative to the other conditions but the average shift was only ~0.5° in the horizontal and ~1° in the vertical direction. Also the variability of eye‐positions and thus the size of the PEP was higher when no visual stimulus was present as shown in Figures 3D and E, while for all other conditions it remained largely the same. To further investigate these effects we conducted three 2‐way repeated measures ANOVAs with the factors “Speed of simulated self‐motion” and “Condition” (Encoding, Reproduction auditory, Reproduction visual, Reproduction bimodal) for the variables “horizontal PEP position,” “vertical PEP position,” and “PEP size.” The factor “Speed of simulated self‐motion” was not significant (*P* > 0.05) for each of the investigated variables, while the factor “Condition” was significant for each (Horizontal position: *F* = 7.1, *P* = 0.04, Vertical position: *F* = 12.7, *P* = 0.01, Size: *F* = 23.4, *P* < 0.01). Post‐hoc t‐tests revealed only significant (*P* < 0.05) differences between the auditory condition and all other conditions for each simulated speed, confirming that the “Reproduction auditory” condition induced different effects from all other conditions.

**Figure 4:**
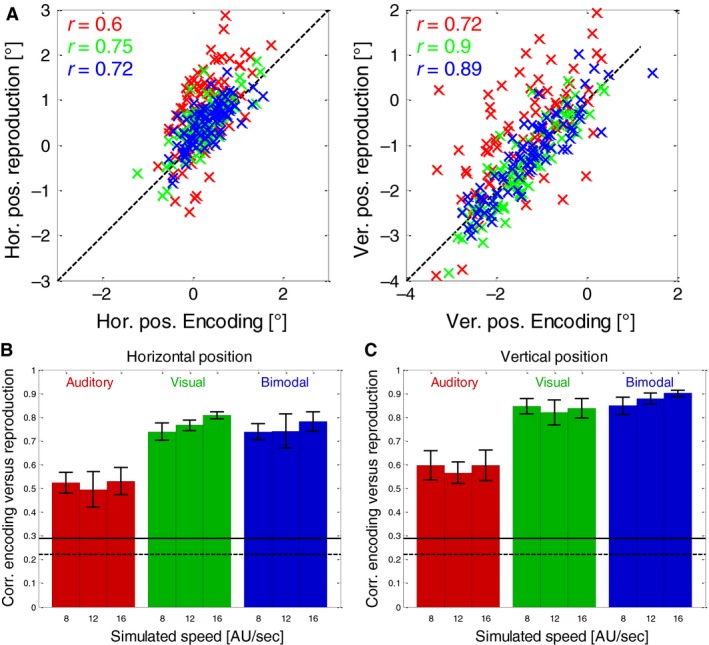
(A) Trial‐by‐trial correlations between the average eye position (horizontal on left panel, vertical on right panel) during encoding and the subsequent reproduction for one example subject (S1) and one example stimulus speed (16 AU/sec). In each condition, 80 trials were performed. Red markers indicate data from auditory, green markers from visual and blue markers from bimodal reproduction. (B) Averages and standard errors (over six subjects) of the correlations between the horizontal eye position during the Encoding and during the Reproduction phases. Different colors indicate which modalities were available during the reproduction. Red bars indicate data from auditory trials, green bars show those from visual trials and blue bars those from bimodal trials. Black horizontal lines indicate the correlation value that was required to reach significance (dashed line, *P* = 0.05; solid line *P* = 0.01) for one subject (based on 80 trials that were performed in each condition). (C) Same conventions as in panel b, but for vertical eye positions.

In the next step, we evaluated the eye positions on trial‐by‐trial basis by averaging the eye‐positions along each spatial dimension during the Encoding and during the following Reproduction phases. Then we calculated a Pearson correlation to determine how much the oculomotor behavior was related during encoding and subsequent reproduction (see example subject in Fig. 4A). We found that when a visual stimulus was present on the screen (visual and bimodal conditions), the eye positions were highly correlated (*P* < 0.001 for each subject and condition, Fig. 4B, C, green and blue bars). Remarkably, this was also the case when only auditory feedback was given during the reproduction (red bars in Fig. 4B and C). Here, the correlations between encoding and reproduction were lower than in cases when visual feedback was present, but still highly significant (*P* < 0.001 in each subject and condition except for one condition in one subject, with *P* < 0.05 and one condition in one subject with *P* > 0.05).

### Eye movements during the encoding and reproduction phases

**Figure 5:**
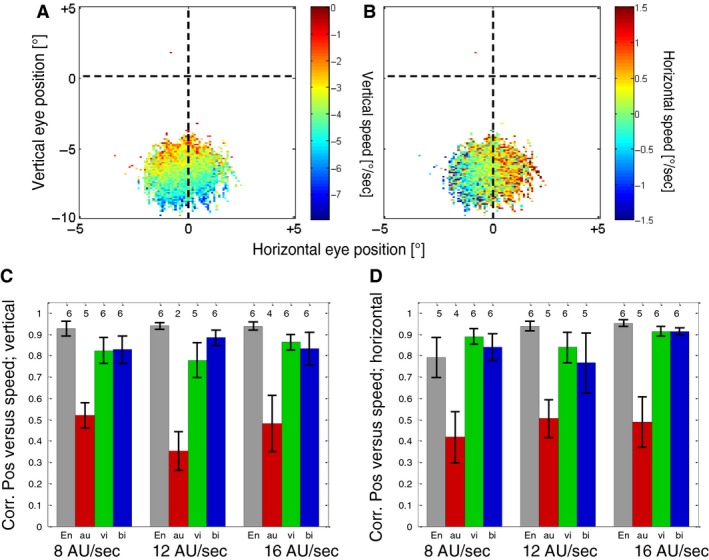
(A) Average vertical speed of the eyes at different positions for one example subject (S4) and condition (Encoding phase, simulated speed of 16 AU/sec). Blue colors indicate fast downward speeds while red and yellow colors indicate slower speeds. (B) Average horizontal speed of the eyes at different positions for the same subject and condition as in 5A. Blue colors indicate data for leftward motion while red colors indicate data for rightward eye movements. (C) Average Pearson correlations between the vertical eye position and vertical eye speed over all subjects. Different bars show the results of different conditions (grey, Encoding phase; red, Reproduction auditory; green Reproduction visual; blue, Reproduction bimodal). Numbers above the bars show the number of subjects (out of six) for whom the correlation was significant (*P* < 0.05). Error bars show SEMs. (D) Average Pearson correlations between the horizontal eye position and horizontal eye speed over all subjects. Conventions are the same as in Figure 5C.

In the next step, we investigated the velocity of slow eye movements during the different conditions of the reproduction task. Figure 5 shows the velocity of the slow phases of eye movements (see Methods) depending on the eye position during the Encoding phase for one example subject. Both the vertical (Fig. 5A) and the horizontal (Fig. 5B) velocities were highly dependent on the position of the eye on the screen and hence the motion vector of the underlying motion stimulus (Fig. 1B). As expected, the downward eye‐velocity increased when the eye position was lower in the display (this is where the vertical stimulus speeds within the stimulus field were highest). The same was true for the horizontal component as shown in Figure 5B, where the direction and the speed of the horizontal component of the eye movements depended strongly on the horizontal eye position. To quantify this relationship, we calculated for each subject a Pearson correlation between the (vertical or horizontal) eye position and the corresponding eye velocity. For the vertical eye movements, we only used the eye positions that were within the area of the visual motion stimulus (*y* < 0°, see Fig. 1A). The results are summarized in Figure 5C and D. They show that when a visual stimulus was present on the screen (Encoding phase, Reproduction visual, Reproduction bimodal), there was a robust correlation between eye position and eye speed in both, the horizontal and the vertical dimensions as expected when the eye movement is driven by the movement of the stimulus. When no visual stimulus was present (Reproduction auditory, red bars), the correlations were weaker, but still significant (*P* < 0.05) in the majority of cases. Interestingly, for one of the subjects the sign of the (significant) correlation reversed in the reproduction auditory condition compared to the visual condition.

**Figure 6:**
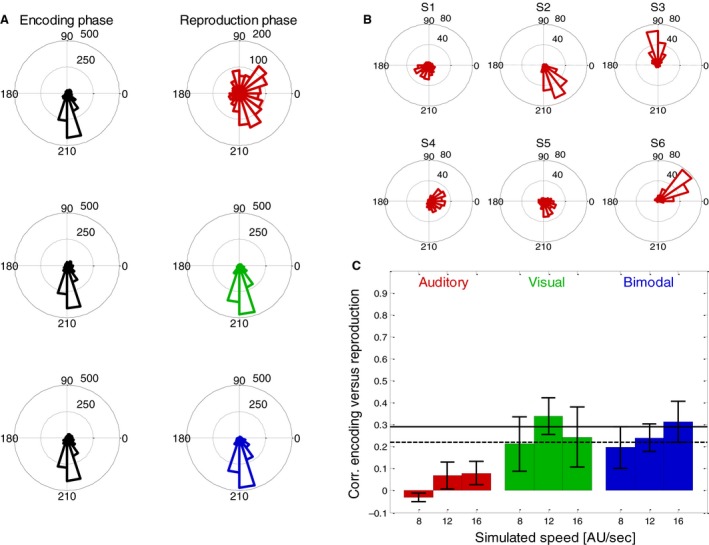
(A) Distribution of average directions of eye movements in all trials of six subjects. The left column shows results from the (always bimodal) Encoding phase and right column results from the different modality conditions (red for auditory, green for visual, blue for bimodal) of the Reproduction phase. (B) Individual distributions of eye movement directions during the Reproduction phase in the auditory condition for each of the six subjects. (C) Averages and standard errors (over six subjects) of the correlations between average motion direction during the Encoding and during the Reproduction phases. Different colors indicate which modality was available during the reproduction. Red bars indicate data from auditory trials, green bars those from visual trials and blue bars those from bimodal trials. Black horizontal lines indicate the correlation that was required to reach significance (dashed line, *P* = 0.05; solid line *P* = 0.01) for each subject (based on 80 trials that were performed in each condition).

When we compared the relative speeds of eye movements in vertical and horizontal directions for the example subject in Figure 5A and B, we found the vertical component to be stronger – which is also a result of the PEPs being placed mostly around the vertical midline (see Fig. 3B), where the horizontal component of the stimulus motion is smallest (see Fig. 1B). Thus, we expected (at least in presence of a visual stimulus) the main component of the eye movements to be downward. This was confirmed when the mean direction of the eye movement was calculated in each trial. Figure 6A shows the distribution of eye movement directions for all conditions. For simplicity, we pooled data from different stimulus speeds and different subjects since the speed had no major effect on the distribution of directions and the subjects were (with an exception that we describe in detail later) quite uniform. As expected, in the presence of a visual stimulus, the main component of the eye movement was downward. The exception is the eye movement during auditory Reproduction phase that shows a broader distribution of directions. When we looked at the results of individual subjects (Fig. 6B), we found that even in the absence of a visual stimulus each subject had a narrow but also very individual distribution of eye movement directions. In the next step, we calculated the circular correlation (Berens 2009) between the mean eye movement direction in the Encoding and the following Reproduction phases. We obtained low correlations in conditions when visual stimuli were present and no significant correlations when a visual stimulus was absent in the Reproduction phase (Fig. 6C).

**Figure 7:**
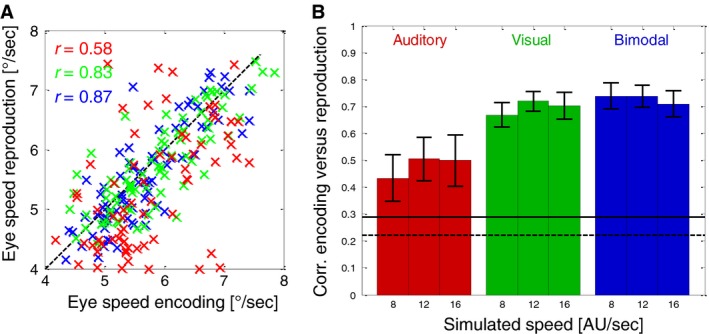
(A) Trial‐by‐trial correlations between the average total eye speed during encoding and the subsequent reproduction for one example subject (S4) and one example stimulus speed (16 AU/sec). The subject performed 80 trials in each condition. Different colors indicate which modalities were available during reproduction: Red markers indicate auditory, green markers visual and blue markers bimodal reproduction. (B) Averages and standard errors (over six subjects) of the correlations between the total eye‐speed during the Encoding and during the Reproduction phases. Different colors indicate which modalities were available during reproduction. Red bars indicate auditory trials, green bars show visual trials and blue bars bimodal trials. Black horizontal lines indicate the correlation that was required to reach significance (dashed line, *P* = 0.05; solid line *P* = 0.01) for one subject (based on 80 trials that were performed in each condition).

From the vertical and horizontal velocity components of the eye movements, we calculated the average absolute speed in each trial, i.e., combining horizontal and vertical components of the eye movements. We then compared the absolute speed of eye movements during Encoding with those during the following Reproduction phases. Figure 7A shows the results for one example subject in all conditions at a simulated speed of 16 AU/sec. It shows significant (*P* < 0.01) correlations for the visual (green marks) and bimodal (blue marks) conditions but as well a somewhat weaker but still highly significant (*P* < 0.01) correlation for the auditory condition. The average correlations for all subjects for all conditions are shown in Figure 7B. Again, visual and bimodal conditions show the highest average correlations while the correlations during auditory reproduction are lower but still clearly significant (significance levels of 5% and 1% are depicted as horizontal black lines in Fig. 7B).

## Discussion

Our results have shown that different aspects of the eye movements in a distance reproduction task correlate between the Encoding and the Reproduction phases. The correlation was weaker ‐ but still highly significant ‐ when no visual stimuli were shown during the Reproduction phase and thus no visual information about self‐motion parameters could be extracted. Our results are consistent with what is known about the role of eye movements in multisensory path integration.

### Eye positions during encoding and reproduction of distance

All our participants have observed a very restricted area of the visual field during the Encoding phase. Since the heading of the stimulated motion was always straight ahead, the main task of the subjects during the Encoding phase was an integration of the self‐motion information to obtain an estimate of traveled distance. Since the speed in the flow field was different at different positions in the visual field, it appears as a useful strategy to utilize a restricted area to reduce the variability of observed visual speeds. If, participants have to estimate the heading for navigation, different parts of the flow field are differently informative. For example, the area around the focus of expansion of the visual flow can become very informative for judgment of heading (Lappe et al. 1999) and the changing positions of this informative area may also be reflected by a higher variability of eye positions. Under natural conditions, factors such as the curvature of a self‐motion trajectory also contribute to the variability of chosen eye positions (Land and Lee 1994; Wilson et al. 2008). A similar behavior as in our experiment was observed at least in a part of subjects during a headway reproduction task in a more naturalistic environment using a driving simulator (Bian et al. 2011). Here, too, as in our study, the task did not involve any heading judgments or requirements for orientation. However, during a real driving task involving steering, it was shown that the subjects covered a much larger area of the visual field with their eye movements (Land and Lee 1994; Crundall and Underwood 1998). This is consistent with the fact that in real driving, not only the speed of the self‐motion but also the heading is important. The changing heading of self‐motion may induce a larger variability of eye movements by generating a larger variability of optic flow. Each of our subjects used a different, idiosyncratic area of the optic flow field during the task. To our surprise, two of our subjects chose an area close to the focus of expansion where the speed of the optic flow was very slow. This may indicate the use of extra‐foveal vision in assessing of the speed of simulated self‐motion.

During reproduction of the previously encoded distance (path integration), the observed area of the visual field did not change. In cases where a visual stimulus was present on the screen, this appears to be a useful strategy as it ensures that the visual motion patterns during reproduction can be compared more easily than those observed during encoding. If only the auditory stimulus was used during reproduction, this behavior appears unnecessary as no visual information can be compared with the encoding. This finding may be interpreted in different ways dependent on whether or not a causal effect of eye movements on path integration is assumed. On the one hand, it can be related to a recent result which shows that eye movements are able to influence the perception of self‐motion even without visual stimuli (Clemens et al. 2017). Thus, the reproduction of the eye movements generated in the encoding phase could be a useful strategy during reproduction to keep this influencing factor constant between the two phases. On the other hand, it may also reflect the functions of memory and imagery in the distance reproduction task.

### Memory and mental imagery during path integration

In the reproduction task, the subjects had to monitor and memorize the distance observed in the Encoding phase and then actively reproduce it. It has been shown that mental imagery is related to visual and auditory memory (e.g., Wheeler et al. 2000) and especially to memory retrieval (Slotnick et al. 2005, 2012; Albers et al. 2013). These previous findings might suggest that mental imagery of visually and auditorily simulated self‐motion information was used in the reproduction phase in particular in cases where no visual information was provided. There is ample evidence showing that visual mental imagery activates areas of the brain that are regularly involved in visual perception (Kosslyn et al. 1995; O'Craven and Kanwisher 2000; Ganis et al. 2004; for a review Kosslyn et al. 2001; Pearson and Kosslyn 2015). In particular, the mental imagery of motion was often shown to activate the same neuronal substrate that is activated during perception of motion (Goebel et al. 1998; Kourtzi and Kanwisher 2000; Emmerling et al. 2016). These motion sensitive areas are also critically involved in the generation of smooth eye movements (Komatsu and Wurtz 1989; for review see: Thier and Ilg 2005). Consequently, it has also been shown earlier that imagery of motion has some of the same consequences as the perception of motion. In particular it is capable of generating a motion aftereffect (Winawer et al. 2010) and slow eye movements (Brown 1968). In line with these previous results, our data show that the (slow) eye movements during the perception of a specific motion stimulus and the recollection/imagery of the same stimulus are strongly correlated.

### Role of after‐nystagmus

Earlier research has shown that a prolonged optokinetic nystagmus is followed by a so‐called optokinetic after‐nystagmus (OKAN, Ohm 1921; Brantberg 1992; Büttner et al. 1976; Cohen et al. 1977) when the subject is placed in complete darkness. OKAN consists of two phases (OKAN I and OKAN II) that are observed at different times after the stimulus is extinguished. In the first phase, the direction of OKAN is the same as the original direction of the nystagmus, while in the second phase the direction is reversed. One may wonder whether the observed eye movements in the absence of the visual stimulus during the reproduction phase might be due to OKAN. We believe that the OKAN did not play a major role in our experiments, since the direction of OKAN in its different phases is either identical to or opposite to the direction of the nystagmus (Büttner et al. 1976). In contrast, the directions of the slow phases of eye movement varied strongly between our subjects (see Fig. 6B). Some of them have even shown directions that were perpendicular to the direction of the original nystagmus which is not consistent with the oculomotor properties of an OKAN.

### Role of eye movements in path integration

Path integration includes the estimation of traveled distance. Mechanisms of path integration were investigated in many different species from insects (Wehner and Srinivasan 1981; Müller and Wehner 1988; Esch and Burns 1996) to humans (Bremmer and Lappe 1999). The main scope of this previous research was the question which internal and external cues are used for path integration and how information from different sources is combined.

During simulated self‐motion, optic flow was identified as one of the important sources of information (bees: Esch and Burns 1996; humans: Bremmer and Lappe 1999; Churan et al. 2017). During active walking without vision, the assessment of path length has been shown to rely on proprioceptive feedback and efference copies. This has been observed, for example in rodents, which update their position much more accurately during actively performed translations than during passive shifts (Mittelstaedt and Mittelstaedt 1982; Etienne et al. 1988). Similar conclusions can be drawn from experiments on normal (Mittelstaedt and Mittelstaedt 2001) and labyrinthine‐defective (Glasauer et al. 1994, 1999) human subjects who had to estimate and reproduce the length of a path or to cover the distance to a previously seen visual target.

During a distance reproduction task, subjects can apply different strategies, such as imitating speed and duration of the Encoding phase. We discussed these strategies in more detail in an earlier publication (Churan et al. 2017) on visuo‐tactile distance reproduction. We concluded that while the possibility for using this strategy cannot be fully discarded, the subjects were able to adjust the duration of individual reproduction trials to account for differences in chosen speed. This indicates that the subjects rather processed an integrated combination of duration and speed than reproducing them independently.

In our experiments, participants were allowed to freely move their eyes. The control of eye movements involves proprioception and efference copies alike (Bremmer et al. 2009; Sun and Goldberg 2016; Zimmermann and Bremmer 2016), also in the context of self‐motion processing (Bremmer et al. 2017). Our results emphasize the role of eye movements and nonvisual processing in path integration and reproduction. This might suggest that oculomotor activity is used as a pacemaker in both distance encoding and reproduction. More research, however, is needed to test this idea and to show to what degree eye movement strategies causally influence performance during path integration. This could be tested, for example by instructing the subjects to fixate a stationary spot during the reproduction task (e.g., Johansson et al. 2012).

## Conflict of Interest

None declared.

## References

[phy213921-bib-0001] Albers, A. M. , P. Kok , I. Toni , H. C. Dijkerman , and F. P. de Lange . 2013 Shared representations for working memory and mental imagery in early visual cortex. Curr. Biol. 23:1427–1431. 23871239 10.1016/j.cub.2013.05.065

[phy213921-bib-0002] Andersen, R. A. , S. Treue , M. Graziano , R. J. Snowden , and N. Qian . 1993 From direction of motion to patterns of motion: hierarchies of motion analysis in the visual cortex Pp. 183–189 in Ono T., Squire L. R., Raichle M. E., Perrett D. I., Fukuda M., eds. Brain mechanisms of perception and memory from neuron to behavior. Oxford University Press, New York.

[phy213921-bib-0003] Angelaki, D. E. , and B. J. M. Hess . 2005 Self‐motion‐induced eye movements: effects on visual acuity and navigation. Nat. Rev. Neurosci. 6:966–976. 16340956 10.1038/nrn1804

[phy213921-bib-0004] Berens, P . 2009 CircStat: a MATLAB toolbox for circular statistics. J. Stat. Softw. 31:1–21.

[phy213921-bib-0005] Berthoz, A. , I. Israël , P. Georges‐François , R. Grasso , and T. Tsuzuku . 1995 Spatial memory of body linear displacement: what is being stored?. Science. 269:95–98. 7604286 10.1126/science.7604286

[phy213921-bib-0006] Bian, Z. , R. Pierce , and G. Andersen . 2011 Eye movement patterns and driving performance. In PROCEEDINGS of the Sixth International Driving Symposium on Human Factors in Driver Assessment, Training and Vehicle Design (pp. 503–509).

[phy213921-bib-0007] Bossard, M. , C. Goulon , and D. R. Mestre . 2016 Viewpoint oscillation improves the perception of distance travelled based on optic flow. J. Vis. 16:4. 10.1167/16.15.4 27919100

[phy213921-bib-0008] Brantberg, K. 1992 Human optokinetic after‐nystagmus: variability in serial test results. Acta Otolaryngol. 112:7–13. 1575040 10.3109/00016489209100776

[phy213921-bib-0009] Bremmer, F. 2011 Multisensory space: from eye‐movements to self‐motion. J. Physiol. 589:815–823. 20921203 10.1113/jphysiol.2010.195537 PMC3060361

[phy213921-bib-0010] Bremmer, F. , and M. Lappe . 1999 The use of optical velocities for distance discrimination and reproduction during visually simulated self motion. Exp. Brain Res. 127:33–42. 10424412 10.1007/s002210050771

[phy213921-bib-0011] Bremmer, F. , M. Kubischik , K.‐P. Hoffmann , and B. Krekelberg . 2009 Neural dynamics of saccadic suppression. J. Neurosci. 29:12374–12383. 19812313 10.1523/JNEUROSCI.2908-09.2009 PMC2787621

[phy213921-bib-0012] Bremmer, F. , J. Churan , and M. Lappe . 2017 Heading representations in primates are compressed by saccades. Nat. Commun. 8:920. 29030557 10.1038/s41467-017-01021-5 PMC5640607

[phy213921-bib-0013] Brown, B. B. 1968 Visual recall ability and eye movements. Psychophysiology 4:300–306. 5636302 10.1111/j.1469-8986.1968.tb02771.x

[phy213921-bib-0014] Büttner, U. , W. Waespe , and V. Henn . 1976 Duration and direction of optokinetic after‐nystagmus as a function of stimulus exposure time in the monkey. Arch. Psychiatr. Nervenkr. 222:281–291. 828038 10.1007/BF00343237

[phy213921-bib-0015] Churan, J. , J. Paul , S. Klingenhoefer , and F. Bremmer . 2017 Integration of visual and tactile information in reproduction of traveled distance. J. Neurophysiol. 118:1650–1663. 28659463 10.1152/jn.00342.2017 PMC5577551

[phy213921-bib-0016] Clemens, I. A. H. , L. P. J. Selen , A. Pomante , P. R. MacNeilage , and W. P. Medendorp . 2017 Eye movements in darkness modulate self‐motion perception. ENeuro 4:ENEURO.0211–ENEURO16.2016. 10.1523/eneuro.0211-16.2016 PMC5263893 28144623

[phy213921-bib-0017] Cohen, B. , V. Matsuo , and T. Raphan . 1977 Quantitative analysis of the velocity characteristics of optokinetic nystagmus and optokinetic after‐nystagmus. J. Physiol. 270:321–344. 409838 10.1113/jphysiol.1977.sp011955 PMC1353516

[phy213921-bib-0018] Crundall, D. E. , and G. Underwood . 1998 Effects of experience and processing demands on visual information acquisition in drivers. Ergonomics 41:448–458.

[phy213921-bib-0019] Emmerling, T. C. , J. Zimmermann , B. Sorger , M. A. Frost , and R. Goebel . 2016 Decoding the direction of imagined visual motion using 7 T ultra‐high field fMRI. NeuroImage 125:61–73. 26481673 10.1016/j.neuroimage.2015.10.022 PMC4692515

[phy213921-bib-0020] Esch, H. , and J. Burns . 1996 Distance estimation by foraging honeybees. J. Exp. Biol.. 199:155–162. 9317542 10.1242/jeb.199.1.155

[phy213921-bib-0021] Etienne, A. S. , and K. J. Jeffery . 2004 Path integration in mammals. Hippocampus 14:180–192. 15098724 10.1002/hipo.10173

[phy213921-bib-0022] Etienne, A. S. , R. Maurer , and F. Saucy . 1988 Limitations in the assessment of path dependent information. Behaviour 106:81–110.

[phy213921-bib-0023] Ganis, G. , W. L. Thompson , and S. M. Kosslyn . 2004 Brain areas underlying visual mental imagery and visual perception: an fMRI study. Brain Res. Cogn. Brain Res. 20:226–241. 15183394 10.1016/j.cogbrainres.2004.02.012

[phy213921-bib-0024] Gibson, J. J. 1950 The perception of the visual world. Houghton Mifflin, Boston.

[phy213921-bib-0025] Glasauer, S. , M. A. Amorim , E. Vitte , and A. Berthoz . 1994 Goal‐directed linear locomotion in normal and labyrinthine‐defective subjects. Exp. Brain Res. 98:323–335. 8050517 10.1007/BF00228420

[phy213921-bib-0026] Glasauer, S. , M. Dieterich , and T. Brandt . 1999 Simulation of pathological ocular counter‐roll and skew‐torsion by a 3‐D mathematical model. NeuroReport 10:1843–1848. 10501518 10.1097/00001756-199906230-00008

[phy213921-bib-0027] Glasauer, S. , M.‐A. Amorim , I. Viaud‐Delmon , and A. Berthoz . 2002 Differential effects of labyrinthine dysfunction on distance and direction during blindfolded walking of a triangular path. Exp. Brain Res. 145:489–497. 12172660 10.1007/s00221-002-1146-1

[phy213921-bib-0028] Goebel, R. , D. Khorram‐Sefat , L. Muckli , H. Hacker , and W. Singer . 1998 The constructive nature of vision: direct evidence from functional magnetic resonance imaging studies of apparent motion and motion imagery. Eur. J. Neuorsci. 10:1563–1573. 10.1046/j.1460-9568.1998.00181.x 9751129

[phy213921-bib-0029] Johansson, R. , J. Holsanova , R. Dewhurst , and K. Holmqvist . 2012 Eye movements during scene recollection have a functional role, but they are not reinstatements of those produced during encoding. J. Exp. Psychol. Hum. Percept. Perform. 38:1289–1314. 22201467 10.1037/a0026585

[phy213921-bib-0030] Komatsu, H. , and R. H. Wurtz . 1989 Modulation of pursuit eye movements by stimulation of cortical areas MT and MST. J. Neurophysiol. 62:31–47. 2754480 10.1152/jn.1989.62.1.31

[phy213921-bib-0031] Kosslyn, S. M. , W. L. Thompson , I. J. Klm , and N. M. Alpert . 1995 Topographical representations of mental images in primary visual cortex. Nature 378:496–498. 7477406 10.1038/378496a0

[phy213921-bib-0032] Kosslyn, S. M. , G. Ganis , and W. L. Thompson . 2001 Neural foundations of imagery. Nat. Rev. Neurosci. 2:635–642. 11533731 10.1038/35090055

[phy213921-bib-0033] Kourtzi, Z. , and N. Kanwisher . 2000 Activation in human MT/MST by static images with implied motion. J. Cogn. Neurosci. 12:48–55. 10769305 10.1162/08989290051137594

[phy213921-bib-0034] Land, M. F. , and D. N. Lee . 1994 Where we look when we steer. Nature 369:742–744. 8008066 10.1038/369742a0

[phy213921-bib-0035] Lappe, M. , and J. P. Rauschecker . 1994 Heading detection from optic flow. Nature 369:712–713. 8008064 10.1038/369712a0

[phy213921-bib-0036] Lappe, M. , F. Bremmer , M. Pekel , A. Thiele , and K. P. Hoffmann . 1996 Optic flow processing in monkey STS: a theoretical and experimental approach. J. Neurosci. 16:6265–6285. 8815907 10.1523/JNEUROSCI.16-19-06265.1996 PMC6579186

[phy213921-bib-0037] Lappe, M. , M. Pekel , and K.‐P. Hoffmann . 1998 Optokinetic eye movements elicited by radial optic flow in the macaque monkey. J. Neurophysiol. 79:1461–1480. 9497425 10.1152/jn.1998.79.3.1461

[phy213921-bib-0038] Lappe, M. , F. Bremmer , and A. V. Van Den Berg . 1999 Perception of self‐motion from visual flow. Trends Cogn. Sci. 3:329–336. 10461195 10.1016/s1364-6613(99)01364-9

[phy213921-bib-0039] Lappe, M. , M. Jenkin , and L. R. Harris . 2007 Travel distance estimation from visual motion by leaky path integration. Exp. Brain Res. 180:35–48. 17221221 10.1007/s00221-006-0835-6

[phy213921-bib-0040] Lutfi, R. A. , and W. Wang . 1999 Correlational analysis of acoustic cues for the discrimination of auditory motion. J. Acoust. Soc. Am. 106:919–928. 10462797 10.1121/1.428033

[phy213921-bib-0041] Mittelstaedt, H. , and M.‐L. Mittelstaedt . 1982 Homing by path integration. Springer, Berlin, Heidelberg Pp. 290–297.

[phy213921-bib-0042] Mittelstaedt, M. L. , and H. Mittelstaedt . 2001 Idiothetic navigation in humans: estimation of path length. Exp. Brain Res. 139:318–332. 11545471 10.1007/s002210100735

[phy213921-bib-0043] Müller, M. , and R. Wehner . 1988 Path integration in desert ants, *Cataglyphis fortis* . Proc. Natl Acad. Sci. USA 85:5287–5290. 16593958 10.1073/pnas.85.14.5287 PMC281735

[phy213921-bib-0044] Niemann, T. , M. Lappe , A. Büscher , and K. P. Hoffmann . 1999 Ocular responses to radial optic flow and single accelerated targets in humans. Vision. Res. 39:1359–1371. 10343848 10.1016/s0042-6989(98)00236-3

[phy213921-bib-0045] O'Craven, K. M. , and N. Kanwisher . 2000 Mental imagery of faces and places activates corresponding stiimulus‐specific brain regions. J. Cogn. Neurosci. 12:1013–1023. 11177421 10.1162/08989290051137549

[phy213921-bib-0046] Ohm, J. 1921 Über Optischen Drehnystagmus. Klin Mbl Augenheilk 68:234–235.

[phy213921-bib-0047] Pearson, J. , and S. M. Kosslyn . 2015 The heterogeneity of mental representation: ending the imagery debate: Fig. 1.Proc. Natl Acad. Sci. 112:10089–10092. 26175024 10.1073/pnas.1504933112 PMC4547292

[phy213921-bib-0048] Royden, C. S. , M. S. Banks , and J. A. Crowell . 1992 The perception of heading during eye movements. Nature 360:583–585. 1461280 10.1038/360583a0

[phy213921-bib-0049] Royden, C. S. , J. A. Crowell , and M. S. Banks . 1994 Estimating heading during eye movements. Vision. Res. 34:3197–3214. 7975351 10.1016/0042-6989(94)90084-1

[phy213921-bib-0050] Slotnick, S. D. , W. L. Thompson , and S. M. Kosslyn . 2005 Visual mental imagery induces retinotopically organized activation of early visual areas. Cereb. Cortex 15:1570–1583. 15689519 10.1093/cercor/bhi035

[phy213921-bib-0051] Slotnick, S. D. , W. L. Thompson , and S. M. Kosslyn . 2012 Visual memory and visual mental imagery recruit common control and sensory regions of the brain. Cogn. Neurosci. 3:14–20. 24168646 10.1080/17588928.2011.578210

[phy213921-bib-0052] Sun, L. D. , and M. E. Goldberg . 2016 Corollary discharge and oculomotor proprioception: cortical mechanisms for spatially accurate vision. Annu. Rev. Vis. Sci. 2:61–84. 28532350 10.1146/annurev-vision-082114-035407 PMC5691365

[phy213921-bib-0053] Thier, P. , and U. J. Ilg . 2005 The neural basis of smooth‐pursuit eye movements. Curr. Opin. Neurobiol. 15:645–652. 16271460 10.1016/j.conb.2005.10.013

[phy213921-bib-0054] von Hopffgarten, A. , and F. Bremmer . 2011 Self‐motion reproduction can be affected by associated auditory cues. Seeing Perceiving 24:203–222. 21864463 10.1163/187847511X571005

[phy213921-bib-0055] Warren, W. H. , and D. J. Hannon . 1990 Eye movements and optical flow. J. Opt. Soc. Am. A 7:160–169. 2299447 10.1364/josaa.7.000160

[phy213921-bib-0056] Warren, P. A. , and S. K. Rushton . 2009 Optic flow processing for the assessment of object movement during ego movement. Curr. Biol. 19:1555–1560. 19699091 10.1016/j.cub.2009.07.057

[phy213921-bib-0057] Wehner, R. , and M. V. Srinivasan . 1981 Searching behaviour of desert ants, genus Cataglyphis (Formicidae, Hymenoptera). J. Comp. Physiol. 142:315–338.

[phy213921-bib-0058] Wheeler, M. E. , S. E. Petersen , and R. L. Buckner . 2000 Memory's echo: vivid remembering reactivates sensory‐specific cortex. Proc. Natl Acad. Sci. USA 97:11125–11129. 11005879 10.1073/pnas.97.20.11125 PMC27159

[phy213921-bib-0059] Wilson, M. , M. Chattington , and D. E. Marple‐Horvat . 2008 Eye movements drive steering: reduced eye movement distribution impairs steering and driving performance. J. Mot. Behav. 40:190–202. 18477533 10.3200/JMBR.40.3.190-202

[phy213921-bib-0060] Winawer, J. , A. C. Huk , and L. Boroditsky . 2010 A motion aftereffect from visual imagery of motion. Cognition 114:276–284. 19853246 10.1016/j.cognition.2009.09.010

[phy213921-bib-0061] Zimmermann, E. , and F. Bremmer . 2016 Visual neuroscience: the puzzle of perceptual stability. Curr. Biol. 26:R199–R201. 26954439 10.1016/j.cub.2016.01.050

